# Toward trustworthy COVID-19 interventions: Building vaccine trust through community-university partnerships

**DOI:** 10.1371/journal.pone.0300872

**Published:** 2024-03-27

**Authors:** Laura A. Bray, Lori L. Jervis, Amanda E. Janitz, Laura Ross, Gloria Tallbull, Timothy M. VanWagoner, the CATCH-UP Vaccines Team

**Affiliations:** 1 Center for Applied Social Research, University of Oklahoma, Norman, Oklahoma, United States of America; 2 Department of Anthropology, University of Oklahoma, Norman, Oklahoma, United States of America; 3 Department of Biostatistics and Epidemiology, University of Oklahoma Health Sciences Center, Oklahoma City, Oklahoma, United States of America; 4 Public Health Institute of Oklahoma, Oklahoma City, Oklahoma, United States of America; 5 Department of Pediatrics, University of Oklahoma Health Sciences Center, Oklahoma City, Oklahoma, United States of America; 6 Oklahoma Clinical and Translational Science Institute, University of Oklahoma Health Sciences Center, Oklahoma City, Oklahoma, United States of America; Swiss Paraplegic Research, SWITZERLAND

## Abstract

Prior research identifies trust as critical to increase vaccine acceptance and uptake. However, few intervention studies have sought to develop or test strategies for bolstering vaccine-related trust. To address this gap, this exploratory study identifies features of COVID-19 vaccine hesitancy interventions that can promote or undermine trust across three interconnected domains: institutional, interpersonal, and product (the vaccine itself). We draw on focus groups (N = 27 participants) with community and university partners involved with hosting COVID-19 testing and vaccine events in underserved Oklahoma communities. Focus groups explored participants’ experiences serving community health needs and elicited feedback on proposed vaccine hesitancy interventions. Proposed interventions included two technology-based strategies (text message reminders and tablet-based testimonials and education) and one dialogue-based strategy (anti-body test interpretation). We find that community partners perceived local universities as trustworthy institutions because of their association with popular sports programs, academic credentials, and proximity, creating opportunities to address vaccine-related distrust through community-university partnerships. The most promising intervention strategies for building interpersonal trust included engaging in one-on-one dialogue and using autonomy enhancing approaches. Finally, interventions that successfully encouraged vaccine trust did so by incorporating personalized health information about individuals’ potential level of protection and susceptibility to the COVID-19 virus. These findings can inform future public health efforts to create trustworthy vaccine hesitancy interventions.

## Introduction

In 2019, the World Health Organization recognized vaccine hesitancy as a top ten threat to public health [1]. The consequences of vaccine resistance and refusal have since been realized on a global scale with the COVID-19 pandemic, as increasing numbers of people question the public health recommendations coming from scientific, medical, and governance institutions [2, 3]. In response, a large body of scholarship has emerged focused on understanding vaccine hesitancy and testing interventions to increase vaccine uptake. This research consistently identifies (dis)trust—defined as a person’s willingness to defer to others on matters beyond their knowledge and power and in ways that can potentially harm them [4]—as a key factor within vaccine hesitancy and refusal, including distrust of the vaccine itself, institutions involved in their development and regulation, and providers responsible for administering the vaccines [5–14]. Yet despite the demonstrated importance of multiple forms of trust for increasing vaccine uptake, few intervention studies focus on developing or testing strategies that can rebuild or repair trust as a means to address vaccine hesitancy [for related work, see 15–17]. As a result, researchers currently know little about what makes an intervention worthy or unworthy of trust to the vaccine hesitant publics. Developing trustworthy interventions is particularly important for underserved and marginalized communities where ongoing experiences of medical mistreatment and neglect contribute to high levels of distrust [16, 18].

This exploratory study examines what makes COVID-19 vaccine hesitancy interventions trustworthy. We report focus group findings from community health organizations and university partners on their experience conducting COVID-19 testing and vaccine events, as well as feedback on several proposed vaccine hesitancy interventions. Through qualitative analysis of these data, we explore features of vaccine hesitancy interventions that can promote institutional, interpersonal, and vaccine trust, as well factors that can undermine trust across these three domains.

### (Dis)trust and the roots of vaccine hesitancy

Research both preceding and following the 2020–23 COVID-19 pandemic consistently identifies (dis)trust as a key factor within vaccine hesitancy and refusal [5–14]. *Vaccine hesitancy* refers to “an attitude of ambivalence regarding vaccines” [7], particularly during the decision-making process [19], with *vaccine refusal* focused on the behavior of declining to vaccinate despite availability. The causes of vaccine hesitancy are multi-faceted and complex, embedded within interpersonal and institutional relationships and shifting over time. Because of the highly specialized and complex nature of modern biomedicine and healthcare, vaccine acceptance requires that the public rely on scientific and medical institutions to act in their best interest, illustrating the critical role of trust for achieving public health goals. As Adhikari et al. [6] explain, “Default asymmetry in information, comprehensibility, and power between the vaccine providers and the vaccine recipients makes the… [recipient] vulnerable as they have to invest some degree of faith in the trusted party.” Survey research consistently finds a strong relationship between trust and COVID-19 vaccine attitudes and behavior, with respondents indicating low levels of trust in traditional media, public health institutions, government, and science less likely to accept or receive vaccines [5, 20–22].

As a sociological concept, *trust* refers to the willingness to defer “to others about something beyond our knowledge and power [and] in ways that can potentially hurt us” [4]. This definition places power differentials and vulnerability at the core of trust relationships. Trust is required because of differential levels of knowledge and power and thus involves a “leap of faith” to overcome knowledge gaps [23]. As a future oriented concept, trust also invokes risk and uncertainty [24]. Placing trust in a person or institution means risking negative consequences if that trust is betrayed, making it “rational” to withhold or withdraw trust in some instances [25]. Finally, as a relational concept, trust is made and remade through lived experience and interactions over time [23]. This means that events, reasons, or information that allow people to suspend or “bracket off” uncertainties to make a leap of faith may change over time [23].

Vaccine hesitancy research examines trust across three primary domains: institutional, interpersonal, and in the vaccine itself [6, 26]. Although conceptually distinct, these three domains intersect in important ways, with institutional aspects often underlying other forms of distrust. *Institutional trust* focuses on the parties responsible for developing, testing, distributing, and regulating vaccines including governments, public health agencies, the healthcare system, pharmaceutical industry, and science itself. In the US, trust in institutions declined during the COVID-19 pandemic [27–29] because of conflicting and confusing official communication [30], the increasing partisan divide over pandemic policies and public health governance [31], and widespread mis/disinformation [32].

Institutional trust lays the groundwork for the development of interpersonal and vaccine trust [23]. *Interpersonal trust* related to vaccines centers on relationships with representatives of institutions, such as doctors, nurses, and public health officials. Despite reduced trust in recent years, doctors and healthcare professionals remain the most trusted sources of vaccine and health information [30]. Because healthcare professionals represent the point of contact between the public and health/medical institutions, these interpersonal relationships hold the potential to rebuild institutional trust more broadly. However, trust relations with healthcare professionals do not always increase vaccine acceptance because many doctors and nurses share the vaccine concerns of the public more generally [33]. Healthcare practitioners also face time constraints that limit their interactions with patients, making it difficult to engage in personalized conversations that can help address vaccine concerns and anxieties [33]. Additionally, those who are poor, uninsured, or medically underserved frequently do not have a regular provider to develop a trusting relationship [34, 35].

In the context of vaccine hesitancy, loss of confidence in scientific institutions and medical providers entails a loss of epistemic trust, or willingness to accept information or knowledge from another party as true and relevant [7]. This loss of epistemic trust in official sources of information damages *vaccine trust*, or trust that vaccines are necessary, safe, and effective [26]. Although vaccine distrust is often attributed to public ignorance (referred to as the “deficit model” in science and health communication), this approach fails to recognize how the vaccine hesitant public understands risk. For example, health officials emphasize vaccine safety based on population-level data and the statistical rarity of severe adverse outcomes. Vaccine hesitant individuals, by contrast, understand vaccine risk in more individualized ways and focus on whether the vaccine is safe for themselves and their children based on personal history and experience [8, 25, 36]. These epistemic mismatches mean that health officials and institutions often fail to effectively address the public’s vaccine concerns and anxieties, focusing instead on the public’s perceived knowledge deficiencies, and thereby deepening distrust.

Scholars emphasize the need for multi-layered interventions to address vaccine related public distrust. Common recommendations include community-engaged approaches, use of trusted messengers, and dialogue-based interventions [37–40]. These approaches make intuitive sense, but more research is needed to understand whether and how they work to increase trust. In this exploratory study, we examine the trustworthiness of vaccine hesitancy interventions to gain insights into how institutions can begin to build or repair trust relations with the public.

## Data and methods

### Study setting: Underserved Oklahoma communities

Oklahoma, a state in the southcentral US, consistently ranks near the bottom of all states for health outcomes and health system performance [41]. The state has low rates of adult vaccine uptake coupled with high prevalence of chronic health conditions that increase risk for infectious disease-related morbidity and mortality [42–45]. During the early emergency period of the pandemic (Jan 2020 through Jul 2021), Oklahoma had among the highest COVID-19 mortality rates in the country (493 deaths per 100,000 population, compared to 372 nationwide) [46, 47]. COVID-19 vaccination uptake has likewise lagged in Oklahoma across the pandemic, reflecting widespread vaccine hesitancy concerns [48]. As of May 2023, 15.3% of Oklahoma adults had received the updated COVID-19 bivalent booster dose, compared to 20.5% of the population nationally [49]. Underserved rural populations are particularly susceptible to severe impacts from vaccine-preventable infectious disease. Oklahoma is a highly rural state, with one third of all residents residing outside of metro areas [50]. Rural Oklahomans are also less vaccinated than their urban counterparts. Less than half (46.7%) of nonmetro residents in the state have completed their initial COVID-19 vaccine series compared to 58.3% in metro counties [51].

### CATCH-UP: Community COVID-19 testing and vaccine events

Data for this study derive from the NIH-funded CATCH-UP project (Community-engaged Approaches to Testing in Community and Healthcare Settings for Underserved Populations, NIH grant number 3U54GM104938-08S1), which provided technical assistance and staffing support for communities to design, organize, and host COVID-19 testing events in rural and underserved areas with limited access to these services. Depending on availability and need, testing services included polymerase chain reaction (PCR), antigen (rapid), and serology (antibody) testing. As vaccines became available, community partners also began arranging for COVID-19 vaccines to be offered at testing events and organizing separate vaccine events.

Community COVID-19 testing events occurred across Oklahoma between December 2020 and April 2023, organized in partnership with the University of Oklahoma Health Sciences Center (OUHSC), the Public Health Institute of Oklahoma (PHIO), and numerous community health organizations. The testing events were made possible because PHIO, an organization founded in 2004, has developed a robust statewide network of health organizations to bridge government, academia, and communities. In 2012, PHIO established the County Health Improvement Organization model and the Oklahoma Primary Health Care Extension System alongside OU Family Medicine. The system was designed to mobilize communities for public and community health action, through community-designed efforts, including academic-community research efforts. To support CATCH-UP events, PHIO provided a total of $75,000 ($2,500 per community health coalition) to purchase supplies and cover startup costs. PHIO adapted a Community Testing Guide from a tribal partner to provide testing site partners with a document to organize their local testing efforts. Community health coalitions also received incentive funds based on the number of events hosted and tests performed. Incentive dollars ranged from $1,250 to $9,500 per event, with a total of $888,000 dispersed. Incentives funds were required to be invested in the community to reduce COVID-19 related morbidity and mortality but had no other restrictions on their use. For example, incentive funds supported after school programming, walking trails and fitness equipment, food interventions for families experiencing COVID-19 isolation, diabetes prevention, and professional development.

Community partners hosted a total of 465 COVID-19 testing and/or vaccine events and performed 8,054 tests (44% antibody, 31% PCR, and 25% rapid). Events occurred in 29 out of Oklahoma’s 77 counties and included African American urban communities in Tulsa and Oklahoma City, rural Tribal communities, and white rural communities throughout the state. In all, 48 total testing site partners or community coalitions took part in organizing testing events. Community partners encompassed a wide variety of organizations, including workplaces, chambers of commerce, Black fraternities and sororities, educational literacy centers, fire departments, federally qualified health centers, and local health departments. Event sites were similarly varied and ranged from churches, schools, community centers, and concert halls to low-income housing complexes, mobile home parks, and parking lots. Many events were organized alongside other services or activities such as social services (e.g., food distribution, back-to-school drives, and dental clinics) or large social events (e.g., Pride Festival, car show, and sports events). In other events, coalitions partnered with local schools, assisted living facilities, and employers to provide services to residents or employees. In coordinating testing and vaccine events, community health partners took on complex and sophisticated roles, operating in many cases as “mini health departments.” This involved emergency preparedness training, managing supply chains, navigating multiple state-mandated reporting and scheduling platforms, learning CDC reporting guidelines, interfacing with the Public Health Lab, coordinating specimen transport, and responding to diverse community needs during outbreaks.

In August 2021, the research team received a supplementary grant award (CATCH-UP Vaccines, NIH grant number 3U54GM104938-09S3), funded through the Rapid Acceleration of Diagnostics–Underserved Populations program (RADx-UP), to design and test interventions to reduce vaccine hesitancy [for the final intervention protocol, see 52]. With a primary aim to inform intervention design, we conducted focus groups with community partners and others involved in organizing or running community testing and vaccine events. Below, we follow the “Consolidated criteria for reporting qualitative research” (COREQ) checklist to ensure transparency in our methods and findings [53].

### Recruitment

All community and academic partners involved with organizing and staffing the CATCH-UP testing and vaccine events were eligible and invited to participate in a focus group. LR, the CATCH-UP director at PHIO, recruited participants through program listservs, virtual meetings, and email outreach. LB, LJ, and LR regularly evaluated the representativeness of the sample and LR extended personalized email and phone invitations to individuals from geographic, racial, and organizational groups that were not well represented in the sample. Recruitment ended once all potential participants had been given multiple opportunities to attend and event registration dropped to consistently low levels (recruitment period: 12/01/2021-03/31/2023). As incentive, participants received a $40 Walmart gift card following data collection.

### Data collection

Data collection took place either in-person or virtually (through Zoom videoconferencing software), depending on participant preference and COVID-19 safety considerations. Three researchers (LB, LJ, and GT) from the OU Center for Applied Social Research, all experienced in qualitative data collection, organized and facilitated the focus groups/interviews. Two researchers were present at each focus group, one acting as the primary moderator and one taking notes and providing technical support. LB (a sociologist and research scientist) and LJ (a medical anthropologist and professor) moderated most of the focus groups/interviews, with GT (a research scientist) primarily serving in a support role.

At the beginning of each focus group, all parties introduced themselves, and researchers explained the purpose and goals of the study before obtaining oral consent from each participant. Focus groups were audio recorded through Zoom or an internal recorder when data collection occurred virtually and using a digital recorder when in-person. Demographic information was collected from participants via an anonymous survey (online or paper, depending on the data collection format) at the conclusion of each focus group. The response rate for the anonymous demographic survey was 74.1%.

During focus groups, a semi-structured interview guide was used to explore (1) experiences organizing, hosting, and running testing and vaccine events, (2) community response to events, including COVID-19 vaccine beliefs and attitudes, and (3) feedback on three proposed vaccine hesitancy interventions intended for implementation at testing and vaccine events. For intervention feedback, we asked participants about potential community response and likely effectiveness of the interventions, logistical or technological obstacles to implementation, and suggested improvements. Interventions varied slightly across focus groups/interviews as the research team refined the strategies and incorporated feedback from participants.

The CATCH-UP Vaccines Team developed the proposed interventions based on disciplinary expertise, prior research, and experience with the testing and vaccine events. Proposed interventions included: (1) text message reminders, (2) tablet-delivered testimonial and educational content, and (3) one-on-one dialogue focused on antibody test interpretation. *Text message reminders* would be sent either before or during events to inform attendees about COVID-19 vaccine availability and encourage vaccination. Different wording options were presented for feedback, including “Your COVID-19 vaccine is reserved for you” and “Remember to get your COVID-19 vaccine at the event today. First, second, and booster doses are available.” *Tablet-delivered testimonial and educational content* was designed to address specific COVID-19 vaccine concerns of community members. Event attendees would first take a short digital survey via tablet to gather information about their vaccine concerns and status. Based on survey responses, attendees would then see tailored testimonial and educational material responding to those concerns. Researchers also presented a sample educational infographic that responded to common vaccine concerns.

*One-on-one dialogue* encouraged vaccination while delivering antibody test results. The intervention included a semi-structured script for testers to use while delivering test results, as well as a visual showing the difference between individuals with high antibodies and no antibodies (an idea that came out of an early focus group). During focus groups, participants were read a sample script for delivering antibody test results that was meant to help standardize the process across testers. Following antibody results interpretation, testers would engage attendees in one-on-one conversation centered on vaccine questions and concerns.

### Sample

Between December 8, 2021 and March 10, 2022, we conducted 7 focus groups with 2–5 participants present in each group. Because of scheduling conflicts, 6 additional individual interviews were completed with participants who were unable to attend a scheduled focus group. In total, 27 people participated in a CATCH-UP focus group or interview. Due to the COVID-19 pandemic, all but two focus groups and interviews took place virtually over Zoom. In December 2021, when social distancing requirements had eased due to relatively low COVID-19 case counts, one in-person focus group took place in a rural community center. One interview occurred over the phone because of issues with poor internet connectivity. Focus groups/interviews ranged from 43 minutes to 2 hours (average of 1.2 hours). All data collection occurred in English.

Most participants (82%) represented community partners who organized and hosted testing events, but we also talked to PHIO staff (7.4%) who served administrative and support roles, OUHSC employees who performed serology testing, and nursing staff who assisted with viral testing and vaccines (11.1%, see Table 1). Reflecting the gender composition of community partners more broadly, women made up most participants (82%), with the remaining 19% identifying as men. Racially, participants identified as 44.4% white, 11.1% American Indian/Alaska Native, 11.1% Black/African American, and 7.4% more than one race. Seven percent (7.4%) identified as Hispanic.

**Table 1:** CATCH-UP Focus group and interview participant demographics (N = 27).

	n	%
**Role**		
Community health partners	22	81.5
PHIO staff	2	7.4
Nurses or OUHSC testers	3	11.1
**Race**		
American Indian/Alaska Native	3	11.1
Black/African American	3	11.1
Asian	0	0
White	12	44.4
More than one race	2	7.4
Unknown/Not Reported^a^	8	33.3
**Ethnicity**		
Hispanic	2	7.4
Non-Hispanic	18	66.6
Unknown/Not Reported^a^	7	25.9
**Gender** ^b^		
Women	22	81.5
Men	5	18.5

^a^ Participant race and ethnicity was collected through an anonymous survey. Missing data primarily reflects the survey response rate (74%) rather than item non-response.

^b^ Because of incomplete survey data, researchers assigned participant gender based on gender presentation, names/pronouns, and researcher (LR) knowledge of individuals.

### Data analysis

Data processing began with verbatim transcription of audio recordings using NVivo auto-transcription services, followed by researcher review and correction of all transcripts for accuracy. We then analyzed the transcripts using NVivo qualitative data analysis software [54]. LB coded the data with regular discussion and input from LJ and GT on themes and analytic categories. Throughout the data collection and coding process, the core research team (LB, LJ, GT) also met weekly with the larger CATCH-UP Vaccines Team to present and discuss preliminary findings and themes. Because of the small sample size and exploratory nature of the study, we did not enlist a second independent coder but rely on other well established means of ensuring analytic rigor, including regular team discussions, transparency in methods reporting, and substantial use of raw data in the findings [55].

**Fig 1:**
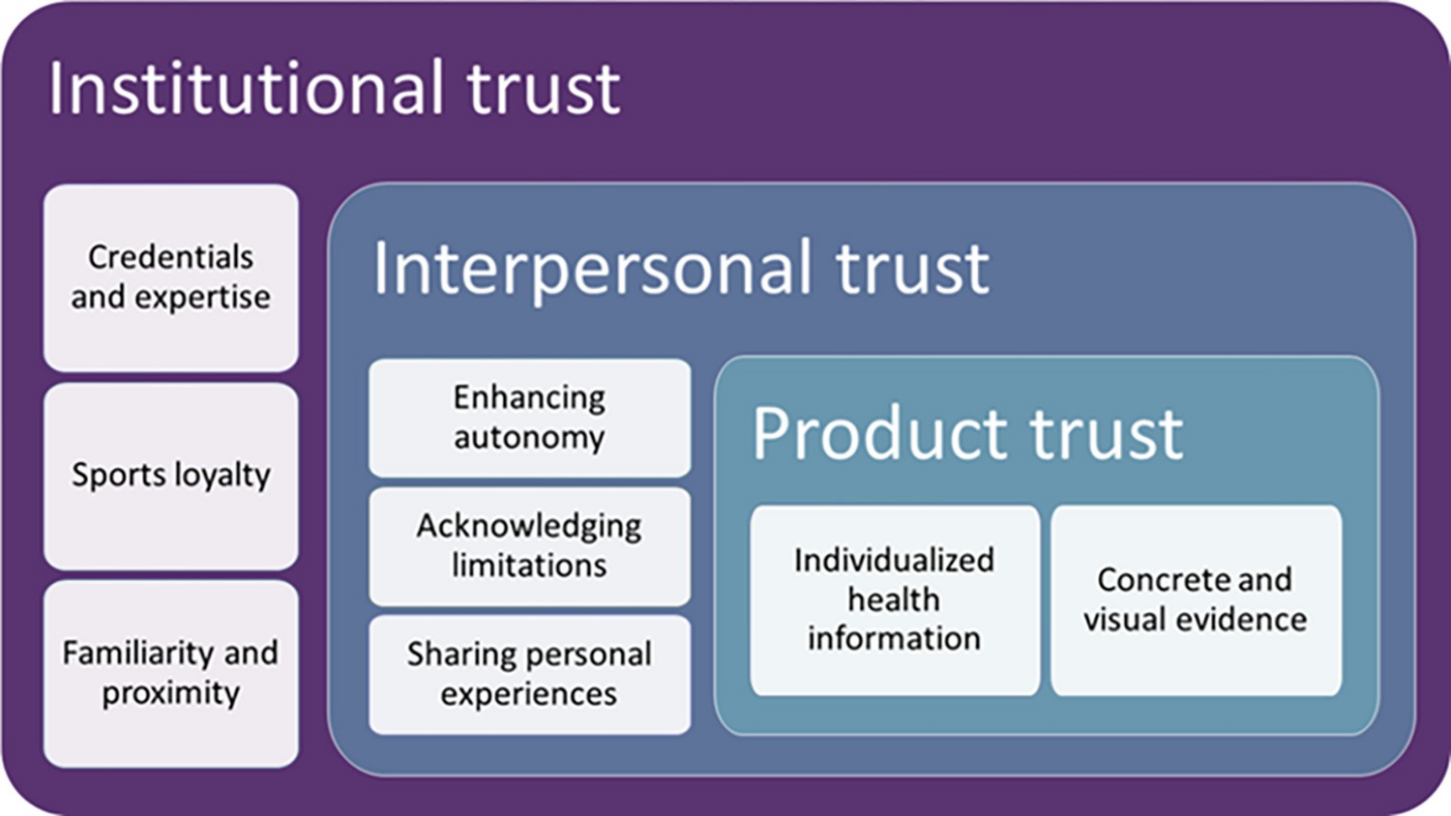
Analytic categories and relationships underlying trustworthy interventions.

Data analysis followed the flexible coding method, a strategy that takes advantage of the capabilities of qualitative data analysis software and recognizes that researchers generally combine deductive and inductive approaches in practice [56]. Flexible coding therefore takes an abductive approach by using previous research to inform data analysis while remaining open to new and unexpected findings [57]. The process involves three stages: indexing, analytic coding, and validity checking [56]. First, we indexed the data by coding transcripts into broad thematic categories based on the interview protocol. Index codes included the organization and success of past testing events, community vaccine attitudes and concerns, strategies used to respond to community vaccine concerns, and feedback on the three interventions. Second, we applied analytic codes to indexed extracts, focused on better understanding how participant strategies and proposed interventions worked to either encourage or reduce trust. Analytic coding combined conceptual categories from past research with inductively developed codes by identifying characteristics of strategies that facilitated trust-building across three pre-determined domains (institutional, interpersonal, and product). Fig 1 presents the analytic codes and their conceptual relationship. Third, we checked the theoretical validity of the coding by examining the distribution of analytic codes across cases, exploring alternative explanations, and identifying negative cases.

### Ethics

The research protocol and materials received expediated approval from the University of Oklahoma Health Sciences Center Institutional Review Board for the Protection of Human Subjects (OUHSC IRB# 13436). We obtained verbal consent from study participants during virtual data collection and written consent during in-person data collection.

## Results

### Institutional trust through community-university partnerships

Community partners identified government and institutional distrust as underlying much of the vaccine hesitancy in their communities and described widespread beliefs that the vaccine had been developed too quickly with experimental technology, was inadequately tested, inferior to natural immunity, and ineffective at preventing infection. Yet despite low levels of trust in national public health and governance institutions, Oklahoma’s large state universities retained some credibility within communities, providing an entry point for community engagement and trust building through community-university partnerships. Working with community partners further bolstered trustworthiness by ensuring the interventions included shared values and identities.

Community health partners attributed the success of COVID-19 community testing and vaccine events in part to the presence of the University of Oklahoma (OU). Participants saw communities as more willing to engage and receive COVID-19 information coming from OU or other large state universities than from large federal agencies (e.g., CDC) and believed that this trust stemmed from the university’s popular sports programs (primarily football), academic and medical credentials, and general proximity/familiarity. One community partner from a small rural community explained how college sports loyalties contributed to institutional trust:

*I think that OU*, *there’s a trust there and I hate to say it*, *simply because of the football…Whether it’s OU or OSU [Oklahoma State University]*, *either one*, *it just seems to be something that people pay more attention to*. (Participant 9, Community Health Partner)

Echoing this sentiment, another community partner suggested that athletic coaches and players from OU or other local teams would be good messengers for health information within their community.

For other participants, the academic and medical credentials of OU made the institution trustworthy to communities. When offering COVID-19 testing services, participants stressed the need to use university branding and OU representatives to establish expertise:

*I think that they [community members] want to see somebody dressed professionally in their OU gear and know that they’re speaking with someone who’s bringing that [expert] knowledge to the table*. (Participant 4, Community Health Partner)

*I did feel*, *even from our first event*, *it was so important to establish the fact [that the University of] Oklahoma*, *OU Health is behind this*. *This is OU Health here*. (Participant 18, PHIO staff)

The perceived expertise behind OU—and particularly OU Health—bolstered the legitimacy of events and enabled community partners to bring trustworthy information to communities. The most commonly noted characteristic that set state academic institutions apart from large national or out-of-state institutions with similar credentials was proximity, suggesting that familiarity increased trustworthiness. Participants frequently stressed that when it came to trusted information sources, “the more local, the better” (Participant 16, Community Health Partner). For this reason, the partnership between OU and local organizations helped to further strengthen the trustworthiness of information.

Community partners emphasized the importance of the community-university partnership for delivering trustworthy information, with the collaboration bolstering the credibility of both parties.

*I think [information from] an organization like OU…and then if it’s filtered through our coalition*, *I think it would help to bring the message home*. *So they would know the information came from an institution that has the data*, *has the brainpower behind it*, *and then maybe we can help to deliver the information*. (Participant 17, Community Health Partner)

This “filtering” of information through local organizations was important because community members tended to place higher trust in peers or community leaders who shared key identity characteristics or values, for example racial or regional identity or religious beliefs. Participants believed that some community members felt more comfortable discussing concerns with non-expert peers with whom they could more easily relate but also wanted assurances that the information came from credible sources. This local translating of information from experts increased the perceived trustworthiness of information. The community-university partnership was, therefore, able to combine institutional reputation and shared local values to help establish trusting relations with a broad range of community members.

Two main issues emerged that threatened to undermine rather than build institutional trust through community-university partnerships: (1) under resourcing events and (2) introducing value conflicts through the research. Many community members had a baseline level of trust in the community-university partnership that made them willing to attend and engage with event staff. However, early lessons suggested the importance of organizational competency and capacity for maintaining and building on this trust. For example, at times too few OU employees were available to staff events, resulting in long wait times and making attendees impatient and less willing to engage in meaningful conversations about COVID-19 and vaccines. This could reduce the perceived competency and trustworthiness of both the community-university partnership and institution more broadly.

The second threat to institutional trust concerned cultural and value conflicts introduced by the research. At testing events, researchers invited attendees to participate in a standardized COVID-19 survey, a required study component as part of the NIH CATCH-UP parent project (NIH RADx-UP) [58]. The survey was administered on a tablet and took about 15 minutes and was completed while participants waited for rapid COVID-19 test results. Community partners described multiple complaints related to the survey that indicated a lack of cultural compatibility between the research instrument and communities, particularly in more conservative rural areas:

*It’s a trust issue*. *I personally think that the survey that went along with this really caused more distrust with people because it had so many questions*, *so many things that really have nothing apparently to do with the COVID itself… I mean*, *we had a lot of people*, *with the gender [question] and the different things*, *[questioning] like*, *“Why are they asking about this for the COVID [study]*?*”* (Participant 22, Community Health Partner)

The gender question refers to a survey item asking about participants’ gender identify that included multiple options outside of the male/female gender binary (e.g., transgender and nonbinary). As alluded to by this exchange, the survey—and the gender question in particular—alienated many community members and made them less likely to engage during events. Community health partners serving rural and conservative areas witnessed multiple attendees become so upset in response to questions related to identity or other topics perceived as irrelevant to COVID-19 that they refused to complete the survey or even walked out. As one tester recalled, “One guy at one of our very first events came back and just dropped the iPad on the table and said, ‘I’m done. This is too woke.’ He said, ‘This is too woke’” (Participant 28, OUHSC Employee). The length of the survey was also a common source of complaints within communities. In many instances, community partners were able to mitigate the impacts, but the negative reactions suggest that the potential of the conflict to damage institutional trust.

### Interpersonal trust through dialogue

The testing and vaccine events, organized and ran through community-university health partnerships, provided effective settings for interpersonal dialogue around COVID-19 and vaccines. Both community partners (many of whom did not have medical or healthcare backgrounds) and OU employees reported facilitating effective conversations about vaccines that led attendees to reconsider their decision and, in some cases, vaccinate at the event. While this was best accomplished via one-on-one dialogue, community health partners also believed technology and other educational aids could play a role as well. Participant strategies for facilitating trustworthy vaccine dialogue included using autonomy enhancing language (such as stressing that vaccinating was attendee’s decision), acknowledging their own knowledge limitations on COVID-19 and vaccines, and sharing personal experiences with the vaccine. Barriers to interpersonal trust through dialogue included the time- and labor-intensive nature of the intervention and lack of knowledge and confidence on the part of some community partners to act as peer educators.

During CATCH-UP focus groups, participants gave feedback on three potential interventions that could be implemented at community health events, including two technology-based interventions (text message reminders and an interactive tablet-based education program) and one dialogue-based intervention. The dialogue-based intervention received the most favorable responses, while the technology-based interventions were seen as most helpful in aiding direct, in-person dialogue. Participants’ assessment emerged from their prior experience engaging in one-on-one vaccine conversation during community testing and vaccine events:

*[T]hat one-on-one discussion about the person individually [works] because then you can go into*, *what’s the reason for not getting vaccinated*? *Do you have any health concerns*? *Really one-on-one works the best for us to get people vaccinated*. (Participant 20, Community Health Partner)

This type of direct, personal interaction was seen as the most promising approach to reduce vaccine hesitancy. However, participants emphasized that conversations must be conducted and approached in an appropriate manner to establish or maintain interpersonal trust.

Communication strategies varied based on participants’ level of comfort, experience, and expertise. OU employees responsible for conducting and explaining the antibody tests described breaking down the science about how the immune system responded to the COVID-19 virus and vaccines and answering questions in a straightforward but nonjudgmental way. Most community partners, although lacking the same level of expertise, were comfortable acting as peer educators to address COVID-19 questions and concerns as well. The most common strategy, both for presenting specific information and as a general logic for organizing events, was to educate attendees by providing factual information while emphasizing individual autonomy and choice:

*I always*, *always say*, *it doesn’t matter to me what personal choice you make*. *It matters to me if you’re making an educated choice*. *And then people are usually open to what you have to say*. (Participant 1, Nurse)

*[My approach] was always*, *regardless of what you decide to do*, *let’s just have the conversation*. *Let’s get the education*. *Let’s figure out what you want to do and then you can make your own choice*. *I’ve never told anybody what to do*. (Participant 3, Community Health Partner)

This approach was seen as vital for breaking down individual resistance and overcoming divisions within the community because of widespread concerns about government overreach and loss of personal freedoms from pandemic restrictions. Within black communities, community partners saw this approach as important because of historical medical abuses (such as the Tuskegee syphilis study) that deprived individuals of autonomy and created widespread distrust in medical institutions. Participants reported that community members were generally receptive to information presented in this way and believed their communities were “still listening” despite the politically polarized and entrenched positions depicted by much of the media.

In addition to the interpersonal trust between the public and event staff, interpersonal trust between community partners, OU employees, and PHIO was crucial for the success of events by increasing their knowledge and confidence as peer educators during events. During CATCH-UP focus groups, community partners reported continuously learning from OU employees and relaying that information to community members.

***Participant 6:*** *We would use the time when we didn’t have anybody*…*[to] carry on conversations [with OU employees] to get our questions answered that people had*…

***Participant 3:*** *[So we could] share that information with other people*.

***Participant 6:*** *So the peer side of that says, “OK, did you get your [test] results?*…*Peer-to-peer, let’s talk about this…” And the facts that we had weren’t so far out there because they were legit from the group. And so I think the peer interaction is huge because… they’re not afraid to ask us questions once they’ve warmed up*… *And knowing the answer, that’s the thing you have to know the answer and not just talk like you know it*.

This example shows how interpersonal trust with OU employees enabled community partners to act as peer educators, as well as how interpersonal trust operated at both the professional and peer levels to address vaccine hesitancy issues. Similarly, community partners noted PHIO staff as a trusted source of information who they felt comfortable contacting and had strong working relationships. Participants also saw transparency about the limitations of their knowledge as important for maintaining community trust. Citing their sources and referring attendees to professionals with greater expertise provided a means to stay within their comfort level while continuing to serve the community.

A second common strategy for both community partners and OU employees was to share their personal experiences with COVID-19 vaccination to help alleviate community members’ fears and concerns.

*One way that we’ve been able to get people to become more accepting of the vaccine at some of these events is giving your own personal experience and being real about it… They usually had a positive reception to that and sometimes changed their minds*. (Participant 12, OUHSC Employee)

*I think that too often we don’t have enough people in the community who look like those who we’re serving*, *who can come and share their experiences and say*, *“Hey*, *you know what*, *I got the vaccine*.*” You know*, *who can kind of talk to them and let them know*, *“Hey*, *it’s OK*, *I’m alive*. *I made it*. *I don’t have an extra arm*.*”* (Participant 7, Community Health Partner)

Community and university partners described sharing both the positive and negative aspects of their vaccine experiences, including uncomfortable and dayslong vaccine side effects. Community partners also saw acknowledging that they too had been hesitant at one time as an important way to build trust by validating the vaccine hesitancy concerns of community members.

The greatest barriers to addressing vaccine hesitancy through interpersonal dialogue was resource limitations and comfort level of community partners to act as peer educators. The time- and labor-intensive nature of personalized conversations made them difficult to implement on a consistent basis when events became busy or were short staffed as described above. These conditions did not allow for the type of trustworthy interpersonal dialogue needed to address vaccine hesitancy. Additionally, several focus group participants, particularly those without healthcare backgrounds, explained that they did not feel comfortable answering COVID-19 or vaccine related questions:

*I have no answers for anyone*, *I want to know too*, *you know…I can’t give answers that I don’t know…because that’s not healthy for any of us*. (Participant 9, Community Health Partner)

*Honestly*, *I didn’t address [vaccine questions] too much because I didn’t feel confident enough in my knowledge to really fully answer those questions*. (Participant 5, Community Health Partner)

Participants suggested regular training and educational opportunities to increase community partners’ COVID-19 knowledge and build skills for navigating difficult conversations, as well as ensuring community partners presented consistent information across the state.

In addition to a lack of confidence in their own COVID-19 and vaccine knowledge, several focus group participants expressed vaccine hesitancy concerns, in some cases leading to vaccine refusal. Expressed reasons for vaccine hesitancy mirrored those of the community more generally and included concerns about long-term health impacts, government overreach, and lack of transparency from pharmaceutical and medical institutions. Notably, vaccine hesitant community partners nonetheless supported and helped to facilitate COVID-19 testing and vaccine events as a means of educating communities—an activity largely seen as neutral and without an ulterior agenda—and because of the financial incentive that their organizations received for hosting events.

### Vaccine trust through individualized health information

By developing institutional and interpersonal trust, community events ultimately sought to increase trust in the COVID-19 vaccine. The dialogue-based intervention proposed to focus groups was built around antibody testing and emerged from earlier events where this service proved unexpectedly popular. Antibody tests used a blood draw (finger prick) to measure the presence or absence of two types of COVID-19 antibodies, IgG and IgM. At testing events, OU employees explained test results to attendees and, time permitting, facilitated a conversation about vaccines. These interactions simplified aspects of immunity for a lay audience and focused on encouraging vaccination rather than providing medical advice. The proposed intervention added a semi-structured script to help standardize the process. Based on experience, participants saw this intervention as the most effective strategy to increase vaccine trust because of its ability to deliver individualized health information in a concrete way and to facilitate vaccine conversations. Specifically, antibody information helped to address questions about personal vulnerability and vaccine effectiveness, both of which represent key factors in vaccine hesitancy [59, 60]. However, antibody testing had the potential to backfire and reduce vaccine trust when community members interpreted results as showing that the vaccines were ineffective or unnecessary. The ability of the tests to deliver new information also varied by individuals and across the pandemic.

For community partners, the antibody testing represented a compelling pathway to transition into providing vaccine services to communities and addressing vaccine hesitancy. Organizers did not initially conceive of antibody testing as a core part of testing events but responded to their popularity and community demand. As one PHIO staff member explained, community partners immediately recognized the opportunity to encourage vaccine acceptance through antibody testing, leading to greater efforts to incorporate COVID-19 vaccine services at events:

*[I]t was once [community organizers] saw that popularity of antibody testing that they really started thinking about vaccines and trying to get vaccines to their events because they felt like they had a clear communication message*. (Participant 8, PHIO Staff)

Unlike other more generic forms of education or viral testing, community partners saw the antibody testing as delivering a clear message about individuals’ potential level of protection and susceptibility to the virus that, in some instances, led them to rethink their vaccine decision. The same PHIO staff member quoted above went on to explain the line of communication:

*A lot of people… would come [to events] and say*, *“Well*, *I had [COVID-19]*, *I’m sure I had it*. *So I have immunity*.*” And you’d be like*, *“Well…*.*let’s test it*.*" If they were negative [for antibodies]*, *they were suddenly questioning*, *“What can I do*? *What should I do*? *Like*, *I’m surprised” in a way that they didn’t do if it was a PCR or rapid test*…*It was a clear message of “I’m susceptible to this disease that I maybe thought I wasn’t susceptible to at this time*.*”* (Participant 8, PHIO Staff)

At a time when many scientific questions remained about the level and duration of protection acquired through different COVID-19 vaccines, as well as disease exposure, the antibody testing was able to provide real-time information that increased people’s confidence in making vaccine decisions.

Several community partners described their own experience with antibody testing and how it informed their vaccination decision:

*I was not vaccinated*. *I had COVID in January last year*. *My doctor said I should be good to go*, *I should have antibodies or whatever*. *I thought I was completely safe*. *The [community coalition]… did an event at the church up here and I went [and tested] and I had no antibodies*. *And that scared me*, *you know*. *And it made me start thinking… maybe I better go get vaccinated*. *And that’s what the antibody testing caused me to do*, *go get vaccinated*. *If I hadn’t done the antibody testing*, *I don’t know if I would be vaccinated today*. (Participant 9, Community Health Partner)

In this case, the participant described how their fear about being unprotected outweighed other concerns about vaccine safety. The antibody test also helped many attendees who had received an initial vaccine series but were undecided about whether or when to get additional shots. One community partner, for example, recalled: “We had a person in our office get tested and she’s on immunosuppressants and she realized that she needed the booster before that was a recommendation” (Participant 16, Community Health Partner). Attendees making decisions about boosters generally expressed less vaccine hesitancy and distrust than their unvaccinated peers. Nonetheless, the antibody testing helped to cut through confusion and conflicting information that could potentially diminish vaccine trust.

In addition to delivering individualized health information, the antibody testing proved appealing for its ability to make the COVID-19 vaccine’s effect on the body and immune system visible and concrete. People were able to “see” the vaccines’ potential for protection at work through the appearance of the bright lines indicating the presence of antibodies in their blood. Community partners discovered that this visibility and concreteness could be enhanced through comparison with other’s (anonymous) test results. One community partner explained, “It was absolutely a turnaround point for us when we could show a comparison. You know, ‘This is somebody who was vaccinated last year. This is somebody who was vaccinated a month ago’” (Participant 6, Community Health Partner). Event staff were then able to explain how vaccines “wore off” over time and the need to update vaccine protection.

The antibody testing provided an important opening for dialogue as vaccine hesitant community members began to question their vaccine beliefs and decisions. However, some community partners reported instances where the antibody testing increased vaccine distrust, as explained by one organizer: “And now the mistrust is higher because… we had people who had been vaccinated and four weeks later got a serology test and didn’t have any antibodies in their system” (Participant 20, Community Health Partner). Test results also had little impact on vaccine trust when they confirmed prior assumptions about protection levels and therefore failed to offer any new information. For example, tests showing that community members had some level of protection from a previous exposure risked reinforcing pre-existing ideas that vaccines are unnecessary and make people less receptive to engaging in further vaccine conversations. In cases such these, the presence of an OUHSC staff member who could explain the science behind such results using lay language was crucial. These examples underscore the importance of dialogue with trusted messengers to accompany antibody testing or other services to avoid unintentionally reinforcing vaccine distrust.

## Discussion

The COVID-19 pandemic revealed widespread public distrust of key societal institutions—including the government, media, and healthcare systems—that fueled vaccine hesitancy and refusal. Trust is a crucial element for increasing vaccine confidence and uptake, yet building trust in a highly polarized context and across demographic differences remains a significant challenge that will require long-term institutional commitment and change. This study reports findings from a community-engaged study centered on COVID-19 testing and vaccine events in underserved Oklahoma communities. We focus on what makes institutions, interpersonal interactions, and vaccines trustworthy. Through focus groups and interviews with community partners, we identify several strategies that successfully facilitated vaccine-related trust. As an exploratory study based on a relatively small sample, this research has inherent limitations regarding generalizability. Future research, some of which is currently in progress by our research team [52], should test interventions that incorporate “trustworthy” characteristics identified in this study to determine their effectiveness at both increasing trust and vaccine confidence. The extent to which our findings apply to distrust and hesitancy surrounding other types of vaccines is also unknown. Nonetheless, our findings offer important lessons for designing and implementing effective vaccine hesitancy interventions.

First, our findings indicate that success of the community-university partnership, including effectively implementing vaccine hesitancy strategies, depended on both institutional and interpersonal trust between partners. The institutional arrangement behind the partnership played a large role in facilitating this trust. PHIO has developed statewide organizational infrastructure and long-term relationships with communities. As a result, the community coalitions hosting and organizing events had pre-existing relationships and experience partnering with OU on health projects. This working relationship provided a foundation of trust between the community and university partners. On an interpersonal level, study personnel spent countless hours working alongside community partners during events developing personal and trusting relationships. These relationships were critical for providing community partners with the knowledge and confidence to act as peer educators.

Second, the cultural resonance of interventions set the stage for trust building. Community partners indicated that community members placed a relatively high level of trust in the university. This trust stemmed in part from its academic and medical credentials, but also from familiarity and the popularity of sports teams. Some saw the university’s association with football and sports as helping to drive attendance at events, as well as increasing epistemic trust. Although popular throughout the country, in the US South, football plays a particularly important role in creating community bonds and collective identity [61]. While some study personnel found the degree of affinity with the University of Oklahoma at odds with previous perceptions that rural Oklahomans viewed the university with suspicion, participants repeatedly reassured us that this was not the case. Our findings suggest that public health campaigns can productively leverage the social and cultural importance of sports to build vaccine trust, for example, by recruiting athletes and coaches to participate in public health campaigns. While sports loyalty alone is unlikely to automatically translate into epistemic trust and survey research indicates low levels of trust in athletes and sports personalities as sources of health information [62], we suggest that it can establish common ground and an entry point for further dialogue and trust building, especially in contexts with high levels of sports enthusiasm and identification with teams. Conversely, cultural misalignment of study components eroded institutional trust.

Cultural misalignment emerged primarily from a survey administered at testing events as required by the NIH RADx-UP parent project. Because the survey was standardized across data collection sites nationwide, the CATCH-UP team had limited control over survey content but did successfully advocate for some small changes in the wording of demographic questions to reduce resistance in Oklahoma communities, while also remaining inclusive of diverse identities. Community partners’ ability to manage cultural conflicts also proved important. Community health partners described successfully using jokes (e.g., “the survey clearly was not written by Oklahomans”) and explaining how the survey research would benefit their communities to ameliorate negative reactions. Additionally, our findings suggest that navigating cultural tensions can be productive when done in the context of pre-existing trust. Participants from one rural health coalition, for example, reported positive experiences providing testing at a Pride event and serving a demographic who they described having little previous interaction.

Third, antibody testing proved capable of increasing vaccine trust through its ability to deliver individualized health information in a visible and concrete way. The concreteness of the test contrasted with the invisibility of the virus and offered evidence of vaccine effectiveness that resonated more strongly than other types of available information. However, the resonance and popularity of the antibody testing can itself be seen as a reflection of institutional distrust, as well as neoliberal policies that shift the responsibility for health from society to individuals [63]. This individualized responsibility encourages people to “do their own research” (consisting primarily of searching the internet) and make decisions based on personal experience rather than turning to expert knowledge or institutions. In allowing people to “see” vaccine effectiveness for themselves, the antibody testing provided a more direct form of evidence, presumably bypassing the need for institutional trust. As seen in the examples described above where antibody testing led to reduced trust, this strategy can easily backfire if not embedded within broader efforts to build institutional and interpersonal trust.

Lastly, we emphasize the importance of mutual (bi-directional) trust and long-term commitments for repairing distrustful relations. Most scholarship focuses on public distrust of institutions, yet researchers and institutions must also be willing to extend trust to communities. Notably, antibody testing remained popular despite official recommendations against its use for assessing immunity against COVID-19 [64, 65]. Although our goal was to facilitate vaccine conversations and testers encouraged vaccination regardless of the results, community members may have still interpreted and used their results in ways that conflicted with the prevailing scientific understanding. Despite these limitations from a strictly scientific perspective, we chose to trust community health partners’ insights on how their communities could best be reached.

## Conclusions

The ability to leverage community partnerships was made possible by years of outreach and collaboration prior to the pandemic. If not for these earlier efforts, it would have been difficult to forge and build these bonds amid a worldwide pandemic. This demonstrates the need to proactively develop and maintain community-university partnerships *before* a crisis ensues. Other innovations arising from this study, e.g., the unexpected effectiveness of antibody testing as a means of concretizing the virus and providing information in a manner that community members could comprehend, demonstrates the importance of identifying ways to better engage in science communication with communities. Also crucial to these efforts is the ability to determine and appropriately utilize institutional authority in ways that benefit communities, which requires understanding what does and doesn’t resonate locally. These findings–the strength of expert-community collaborations, the importance of developing community relevant science communication, and the ability to effectively understand and deploy institutional authority–provide a roadmap for an improved response in future pandemics and community crises.
